# Design and experiment of a bionic drag-reducing digger for tuberous crops under heavy soil conditions

**DOI:** 10.1371/journal.pone.0318526

**Published:** 2025-02-25

**Authors:** Ranbing Yang, Wenjian Xu, Zhiguo Pan, Huan Zhang, Zhixi Deng

**Affiliations:** 1 College of Mechanical and Electrical Engineering, Qingdao Agricultural University, Qingdao, China; 2 School of Mechanical and Electrical Engineering, Hainan University, Haikou, China; Borlaug Institute for South Asia-CIMMYT, INDIA

## Abstract

Aiming at the problems of high working resistance and high energy consumption in potato crop harvesting in sticky soil, this paper designs a potato bionic drag-reducing digging shovel based on the streamline shape of catfish head. Based on the theoretical analysis and discrete element method (DEM) simulation, the main factors affecting the digging resistance are the angle of entry, forward speed and vibration frequency, and the digging resistance increases with the increase of forward speed, and decreases with the increase of vibration frequency. Through the orthogonal test in the field, the optimal working parameters of the drag reduction performance are determined with the digging resistance as the test index, and the comparative test of the different shovel shapes is carried out with this parameter. The results show that the optimal solution is to use bionic shovel, with an entry angle of 15°, an operating speed of 0.27m/s, and a vibration frequency of 6Hz. The average digging resistance of the bionic shovel is 3612.86N, and the bionic digging shovel reduces resistance by 17.76% relative to an ordinary flat shovel, and 21.09% relative to the plane triangle shovel. The effect of drag reduction is remarkable, and the structure of the digging shovel bionic is reasonable, which can satisfy the requirements of resistance reduction and consumption reduction of potato harvesting under the conditions of sticky and heavy soils.

## Introduction

Heavy soils have better fertiliser retention, and the efficient food production model of rice-potato rotation has also fully driven the cultivation of potato crops in heavy soils, which is conducive to reducing the amount of chemical fertiliser and improving ecological efficiency [1–3]. However, the compact molecular structure of clay-heavy soils often leads to problems such as excessive work resistance and increased energy consumption [4–6].

The rise of bionics brings a brand-new solution to the drag reduction problem of soil-touching components, and experts and scholars at home and abroad conduct a lot of research on the above problems. Liu et al. designed a bionic furrow opener through the surface structure of earthworm head, and analyzed the effect of digging resistance of the bionic furrow opener by discrete element method. The results showed that the bionic furrow opener can reduce the working resistance and improve the quality of furrow opening compared with the ordinary furrow opener [7]. Du et al. researched the surface structure of fish scales and created a similar bionic surface to reduce the resistance by coating technology. The results showed that the bionic structure effectively reduced the forward resistance under the influence of the surface appearance [8]. Li et al used earthworm and scallop as bionic prototypes to design a bionic corrugated structure for drag reduction digging shovels, and the results of the simulation tests showed that the bionic longitudinal corrugated shovels exhibited superior drag reduction performance [9,10].

Secondly, Zou Xiangxiang designed a cassava digging shovel to imitate the front claw digging shovel of oriental mayflies, designed the bionic digging shovel by polynomial curve fitting, and compared and analysed it with ordinary digging shovels, and the results showed that the bionic digging shovel had less digging resistance [11]. Shi Linrong bionic design of mayfly front claw through bionic technology, using curve fitting to establish the fitting equation, design potato bionic digging shovel, through simulation, the results show that the bionic shovel piece than ordinary shovel piece of soil resistance is reduced [12]. Fan Yu used the principle of bionics to complete the data acquisition of wild boar arching mouth, surface reconstruction, extract the characteristic curve, and generate the curve fitting equation. The potato bionic digging shovel is designed and tested by simulation. The results show that the bionic digging shovel has low working resistance and high soil particle crushing rate [13]. Ma Weinan et al. used three-dimensional scanning technology to bionic data acquisition of mole front paw to design windproof bionic digging shovel, which was verified by simulation and soil trench test. The results show that the bionic shovel has better performance of drag reduction and soil crushing [14]. Qiao Yitao designs the bionic surface of the shovel excavation component by fitting equations to the inner and outer contours of the claw toes of the pangolin. The results show that the drag reduction rate of the bionic excavation shovel is 16.5%. The bionic design effectively reduces the working resistance of the shovel excavation component [15].

In summary, bionics shows excellent drag reduction performance in the excavation of subsoil crops. In this paper, on the basis of planar vibration digging shovel, a bionic drag reduction digging shovel for potato for heavy soils is proposed. The bionic design of potato digging shovel is carried out by curve fitting the streamlined structure of catfish head. The front curved lines, ridges, and weft structures are introduced to the digging shovel face. While increasing the crushing effect, the digging shovel’s ability to enter and pass through the soil is enhanced. Reduces drag and consumption in the potato digging process.

## Materials and methods

### Design of a digging shovel modelled on the head of a catfish

Catfish, which are aquatic creatures that live in mud, inhabit underwater sludge in rivers, lakes, ponds, and swamps. Over a long period of natural evolution and selection, catfish have developed a unique advantage in their physical structure. Their heads feature a convex, flat, and streamlined shape, which facilitates their movement through soil. When drilling through mud, catfish use their body swing along with the specific design of their head structure to reduce resistance. This makes it easier for them to bore into the sludge and navigate through it. Their strong ability to enter and move through soil aligns well with the drag reduction needs of potato harvesting processes in heavy soil conditions. The demand for drag reduction in potato harvesting process under soil conditions is the same, so the streamlined structure of catfish head is imitated.

Compared with the plane shovel, the convex bionic digging shovel imitating the streamline shape of the catfish head is more conducive to make the soil stress change during the process of soil crushing, and the soil around the potato is more likely to reach the rupture state, so as to achieve the effect of drag reduction and have a good transition effect on the soil [16].

**Fig 1:**
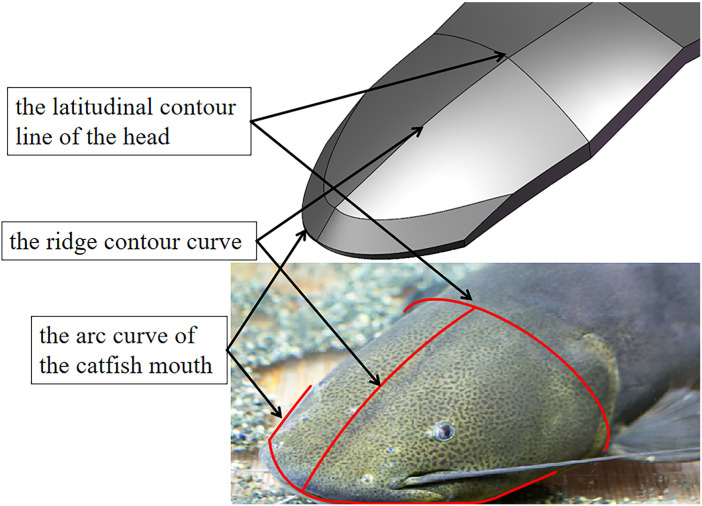
Distribution of the fitting curve position for the digging shovel.

Flat shovels have low passability and higher working resistance under clayey soil conditions [17]. The streamlined structure of the digging shovel modeled after a catfish head allows the soil to pass through with greater shear force as well as less positive stress conditions. At the same time, the convex structure is more favorable to the crushing effect on the soil compared to the flat shovel. It facilitates the entry of the shovel surface into the soil and the passage of the shovel body in heavy and sticky soils [18]. The bionic excavation shovel is obtained by extracting the arc curve of the catfish mouth, the ridge contour curve, the latitudinal contour line of the head and the corresponding surface bionic optimisation. The key points are extracted and the curve is fitted by origin, and the corresponding bionic curve position of the bionic shovel is shown in Fig 1.

The curved curve of the catfish mouth was optimised as a curved entry shovel blade at the front end of the shovel, and the equation fitted to the edges of the entry shovel blade was:


$$y_{1}=4.617\times 10^{-7}{x_{1}}^{3}-0.02357{x_{1}}^{2}+0.0505x+59.4986(R=0.9998)$$ (1)

The value range of *x*_1_ is [−43.2, 43.2], and the chamfering operation is carried out at the tip of the shovel to reduce the thickness of the shovel blade and increase the pressure into the soil, in order to reduce the resistance of the bionic excavation shovel when the shovel surface enters the soil, and to reduce the congestion of the equipment, which is useful for reducing the rent and increasing the efficiency.

The bionic excavating shovel is vertically set up with ridges in the center, which are generated by fitting the contour curve of the ridges of the catfish head, and the fitting equation is:


$$z_{1}=1.05483\times 10^{-5}{x_{2}}^{3}-0.0026{x_{2}}^{2}-0.09149x_{2}-0.24581\left(R=0.9997\right)$$ (2)

The value range of *x*_2_ in the formula is [0, 100], the ridge line as the raised part of the bionic shovel, through the structure of high in the middle and low on both sides, so that the soil passes from both sides of the shovel surface, which can achieve a better effect of crushing the soil. The curved structure of the transition of the potato-soil mixture also plays a role in promoting the transition of the potato-soil mixture.

The weft line was set transversely in the middle, which was made by fitting the weft line contour line of the catfish head, and the fitting equation of the weft line was:


$$z_{2}=1.63596\times 10^{-6}y^{3}-0.01158y^{2}+0.10168y+25.64976(R=0.9940)$$ (3)

**Fig 2:**
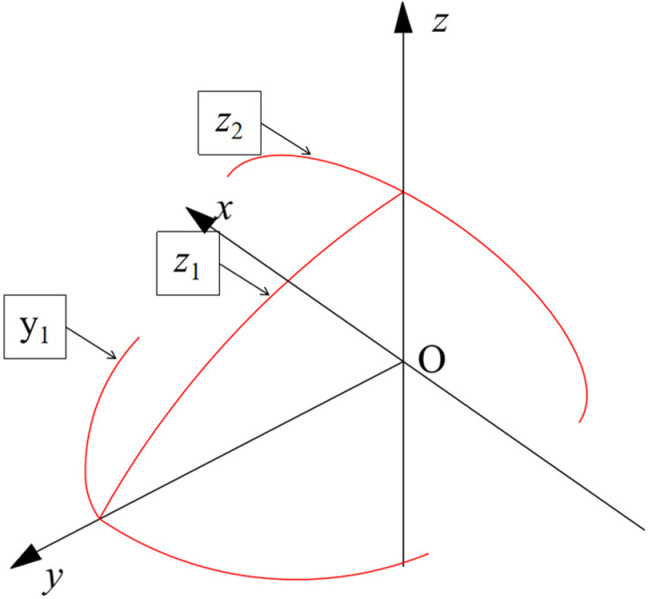
Schematic representation of the position of the fitted curve in the coordinates.

Where *y* is in the range of [−50, 50]. The bionic excavation shovel gradually descends from the ridge line to both sides in the horizontal direction, and gradually raises in the vertical direction from the front end of the entering shovel blade backward until the latitude line, and breaks up the soil through the ridge line and the latitude line in order to achieve a strong soil breaking capacity. The three fitted curves are shown in position Fig 2.

**Fig 3:**
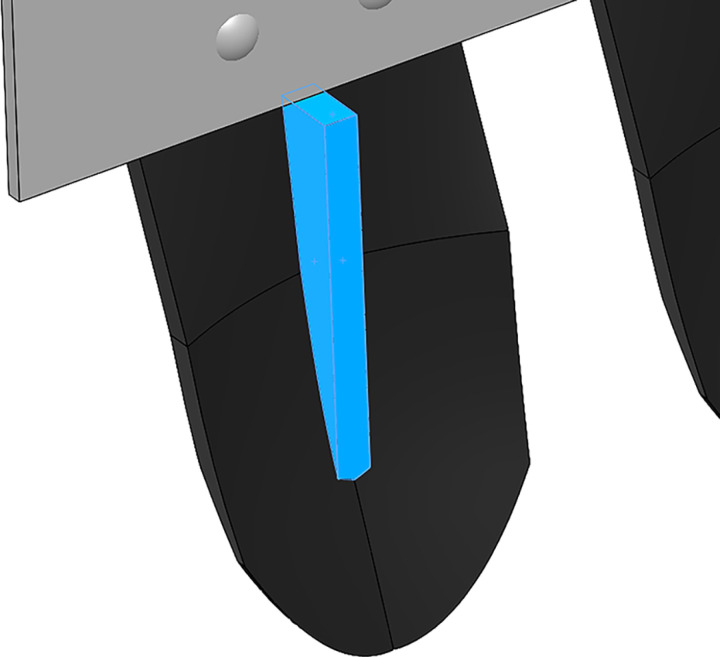
Triangular support plate.

**Fig 4:**
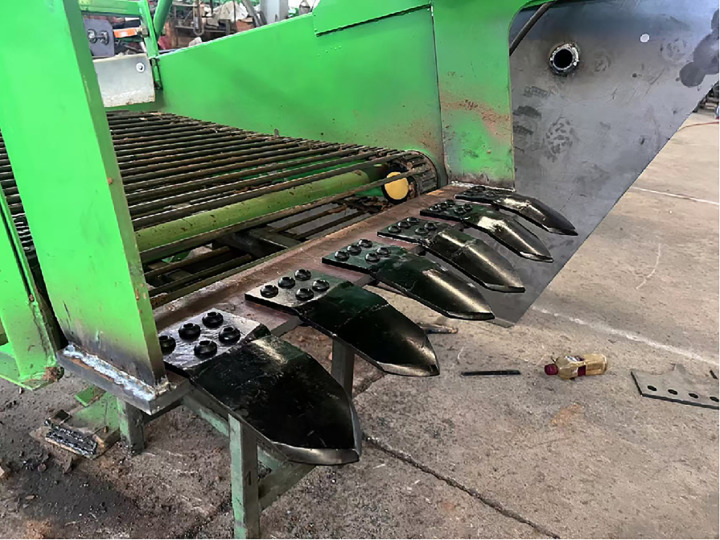
Overall installation.

In the design of shovel body size, the single size of bionic digging shovel is 230mm × 100mm × 8mm, the edge angle is 45°, and the thickness is 8mm. the class triangular support plate is added at the bottom and the connection of the mounting plate in order to enhance the bending resistance of the digging shovel, as shown in Fig 3. The material chosen is 65Mn to increase wear resistance. There are 6 of them evenly arranged, with a total width of 900mm, connected by round head bolts. The bolts are arranged in two rows with 2 in front and 3 in the back to cope with the positive pressure and shear force caused by vibration excavation. The round head of the bolts also plays a role in breaking up the soil, and the physical drawing of the whole device is shown in Fig 4. The digging shovel entry section is connected to the connecting section by a transition, and the arrangement spacing is set to 40 mm. 40 mm is calculated to be a suitable spacing for the soil to pass through and for the potato pieces to be unable to pass through. When the potato soil mixture passes through the digging mechanism, part of the soil is filtered through the gap. The potato-soil mixture passing through the digging mechanism is reduced, the load of the digging mechanism is reduced, and the digging resistance of the mechanism is reduced. Favorable working conditions are provided for the screening of the conveyor chain.

## Results and analysis

### Bionic excavating shovel force analysis

**Fig 5:**
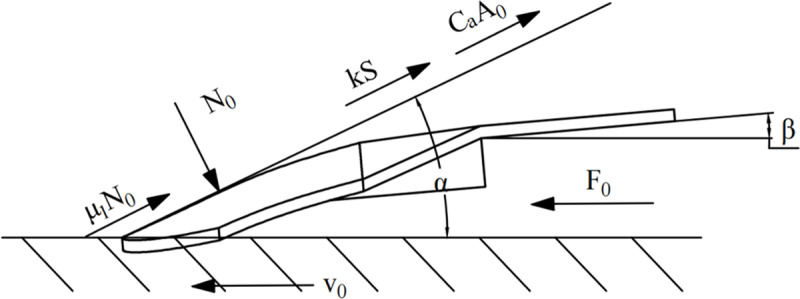
Shovel face force analysis diagram.

In the potato harvesting process, the digging shovel serves as the main force-receiving component, therefore, the strength of the digging shovel and the drag reduction performance of the digging device are the main factors for performance assessment [19]. The forces on the digging shovel are analyzed theoretically. The working resistance mainly originates from the traction force of the machine, the positive pressure of the soil accumulation on the shovel surface. The frictional resistance of the soil to the shovel surface as it passes over the shovel. Heavy soil on the shovel surface adhesion generated by the shovel surface adhesion, and digging shovel cutting resistance to the soil. Combining the above various external forces on the bionic shovel surface for force analysis, as shown in Fig 5.

Force analysis of the excavation shovel in the horizontal direction


$$F_{0}-N_{0}\sin\alpha -(\mu_{1}N_{0}+kS+C_{a}A_{0})\cos\alpha =0$$ (4)

where *F*_0_ -- traction resistance/digging resistance, N, *N*_0_ -- normal load of digging part, α -- shovel surface inclination angle, (°), *μ*_1_ -- coefficient of friction between soil and metal, *k* -- pure cutting force, N, *S* -- width of excavating shovel, mm, *C*_a_ -- adhesion factor between soil and shovel, N/m^2^, *A*_0_-- excavation shovel surface area, mm^2^.

The pure cutting force exists only when there are more stones or when the shovel blade is blunt, and the clay soil conditions have fewer stones in the soil, so the pure cutting resistance of the excavating shovel can be neglected [20], i.e., the simplified formula for the traction force without pure cutting force is


$$F_{0}=N_{0}\sin\alpha +(\mu_{1}N_{0}+C_{a}A_{0})\cos\alpha$$ (5)

According to Newton’s third law, the total resistance of the soil is


$$F=F_{0}$$ (6)

**Fig 6:**
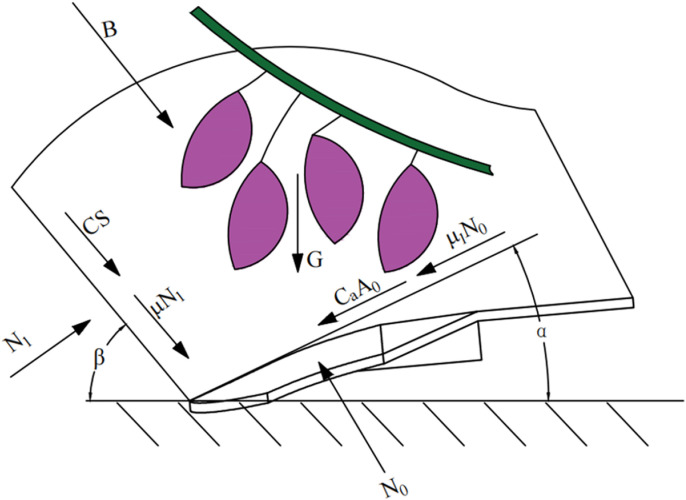
Force analysis of potato soil mixture.

The potato-soil mixture acting on the shovel face of the digging shovel is analyzed. The gravity of the potato soil mixture, the acceleration force of the soil moving along the shovel surface, the support force of the digging shovel on the soil, the reaction force of the frictional resistance, the reaction force of the adhesion force, the normal load of the front failure surface, and the internal friction force of the failure surface. The force analysis of the potato soil mixture by combining the above external forces is shown in Fig 6.

Force analysis in the horizontal direction


$${\text{N}}_{0}\sin\alpha +\mu_{1}{\text{N}}_{0}\cos\alpha +{\text{C}}_{\text{a}}{\text{A}}_{\text{0}}\text{cos}\alpha -N_{1}\sin\beta -(B+CS+\mu N_{1})\cos\beta =0$$ (7)

Force analysis in the vertical direction


$$G-{\text{N}}_{0}\cos\alpha +(\mu_{1}N_{0}+C_{0}A_{0})\sin\alpha -N_{1}\cos\beta +(B+CS+\mu N_{1})\sin\beta =0$$ (8)

where *C* -- unit area soil cohesion, N/m^2^, *N*_1_--normal load on the front failure surface, N, *β*--Front failure inclination angle, (°), *B* - acceleration force per unit area of soil, N, *S* - soil shear area, m^2^, *μ* - soil internal friction coefficient.

Equations (6)–(8) are united to obtain N_0_ which is simplified by bringing (5):


$$F(\frac{\cos\alpha -\mu_{1}\sin\alpha }{\sin\alpha +\mu_{1}\cos\alpha }-\frac{\mu \sin\beta -\cos\beta }{\sin\beta +\mu \cos\beta })=G+\frac{B+CS}{\sin\beta +\mu \cos\beta }+\frac{{\text{C}}_{\text{a}}{\text{A}}_{\text{0}}}{\sin\alpha +\mu_{1}\cos\alpha }$$ (9)

Let


$$Q=\frac{\cos\alpha -\mu_{1}\sin\alpha }{\sin\alpha +\mu_{1}\cos\alpha }-\frac{\mu \sin\beta -\cos\beta }{\sin\beta +\mu \cos\beta }$$ (10)

Follow


$$F=\frac{G}{Q}+\frac{B+CS}{Q(\sin\beta +\mu \cos\beta )}+\frac{{\text{C}}_{\text{a}}{\text{A}}_{\text{0}}}{Q(\sin\alpha +\mu_{1}\cos\alpha )}$$ (11)

Through the force analysis of the bionic digging shovel and potato soil mixture, it can be seen that the resistance of the digging shovel is mainly affected by the digging shovel geometric structure parameters, physical properties of the soil parameters, operating parameters and other factors, the digging shovel’s angle of entry, the forward speed and vibration frequency are the main factors affecting the resistance of digging.

Calculus derivative of force F by shovel surface inclination angle and Front failure inclination angle.


$$\{\begin{array}{l} \frac{\partial F}{\partial \alpha }=-\frac{C_{a}A_{0}}{Q{\left(\sin\alpha +\mu_{1}\cos\alpha \right)}^{2}}(\cos\alpha -\mu_{1}\sin\alpha )=0\\ \frac{\partial F}{\partial \beta }=-\frac{B+CS}{Q{\left(\sin\beta +\mu \cos\beta \right)}^{2}}(\cos\beta -\mu \sin\beta )=0\\ \left(0\leq \alpha \leq \frac{\pi }{2},0\leq \beta \leq \frac{\pi }{2}\right) \end{array}$$ (12)

solve for


$$\{\begin{array}{l} \alpha =\arctan(\frac{1}{\mu_{1}})+k\pi ,k\in Z\\ \beta =\arctan(\frac{1}{\mu })+k\pi ,k\in Z \end{array}$$ (13)

From equations (12) and (13), it is evident that as *α* and *β* converge towards 45°, the derivative of the digging resistance *F* approaches zero, indicating a minimal digging resistance. Literature review indicates that the entry angle *α* ranges from 10° to 20°, while the inclination angle *β* of the front failure surface ranges from 28° to 35° [21]. By analyzing the influence curve of different entry angles on digging resistance, in conjunction with the design parameters of typical potato digging shovels, we have determined that an entry angle of 15° for the bionic shovel is optimal.

### Discrete element simulation test

#### Simulation test design.

In order to test the feasibility of the bionic excavation device, based on the discrete element method, the simulation software EDEM is used to create a model of the digging shovel and soil simulation operation. The simulation test of the bionic digging shovel is mainly simulated and analyzed on three aspects: different shovel shapes, forward speed, and different vibration frequencies. Explore the effect of different working conditions on the digging resistance.

The specific methods are as follows: in order to consider the influence of digging shovel size on the test results, the size of the plane shovel is determined to be the same as the overall size of the bionic shovel. Different forward speeds are selected to simulate the bionic digging shovel, and different vibration frequencies are simulated to have an effect on the digging resistance, and in terms of the choice of shovel shape, the existing ordinary flat shovels, planar triangular shovels and self-developed bionic shovels are selected to carry out a comparative test of different shovel shapes. In terms of shovel shape selection, the existing ordinary flat shovel, planar triangular shovel and self-developed bionic shovel are selected for the comparison test of different shovel shapes.

**Fig 7:**
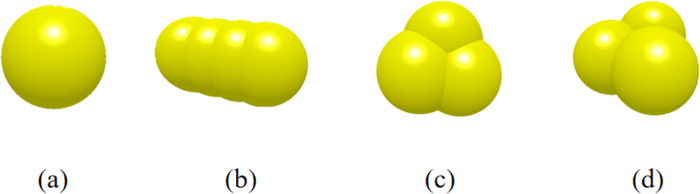
Soil particle model. (a) A single spherical; (b) Columnar; (c) Compact triangular; (d) Loose triangular particles.

In order to simulate the complexity of the heavy soil environment, the particle model of the soil is set up, the soil with higher water content will appear a certain bonding effect. Compared with the Hertz Mindlin (no slip) model is mostly used for non-viscous, incompressible scenarios, the Hertz Mindlin with JKR is often used to deal with viscous, incompressible scenarios, which is more suitable for simulation and analysis of heavy soils, so the JKR model is selected [22]. for the simulation of heavy soils particles to establish a single spherical, columnar, compact triangular and loose triangular particles of four different shapes to respectively 25% of the proportion of the composition of the simulation of the soil model. The four pellets have a single ball diameter of 5 mm, as shown in Fig 7 [23]. the material parameters and contact parameters as shown in Table 1 [9].

**Table 1:** Simulates material parameters and contact parameters.

Parametric	Numerical value
Soil particle density/(kg·m^−3^)	2500
Shovel density/(kg·m^−3^)	7850
Poisson’s ratio for soil	0.35
Poisson’s ratio of shovel body	0.30
Soil shear modulus/(Pa)	1 × 10^6^
Shear modulus of shovel body/(Pa)	7.85 × 10^10^
Coefficient of static friction between soils	0.83
Coefficient of rolling friction between soils	0.25
Coefficient of recovery between soils	0.66
Coefficient of static friction between soil and shovel body	0.56
Coefficient of rolling friction between soil and shovel body	0.18
Coefficient of recovery between soil and spade	0.60
Cohesion between soils (J·m^−2^)	7.91
Cohesion between soil and shovel (J·m^−2^)	6
JKR-Surface Energy (J·m^−2^)	14.88

The particle plant for the simulated motion was tested using an earth slot with a length × width × height of 1000 mm × 900 mm × 300 mm, During the soil trough generation, the soil particles are generated at a rate of 20,000 per second, the total number of generated particles is 100,000, the particle generation time is 5s, the excavation shovel starts moving at 5s, and the simulation time step is 0.01s. The excavation shovels were modelled using a 3D modelling software and imported into the EDEM.

**Fig 8:**
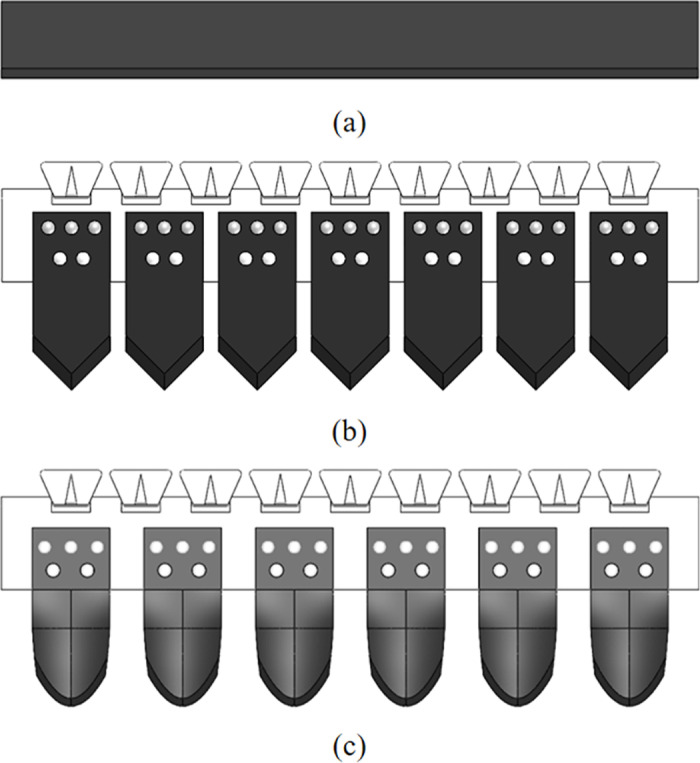
Excavator shovel model. (a) Ordinary flat shovel; (b) Plane triangular shovel; (c) Bionic shovel.

**Fig 9:**
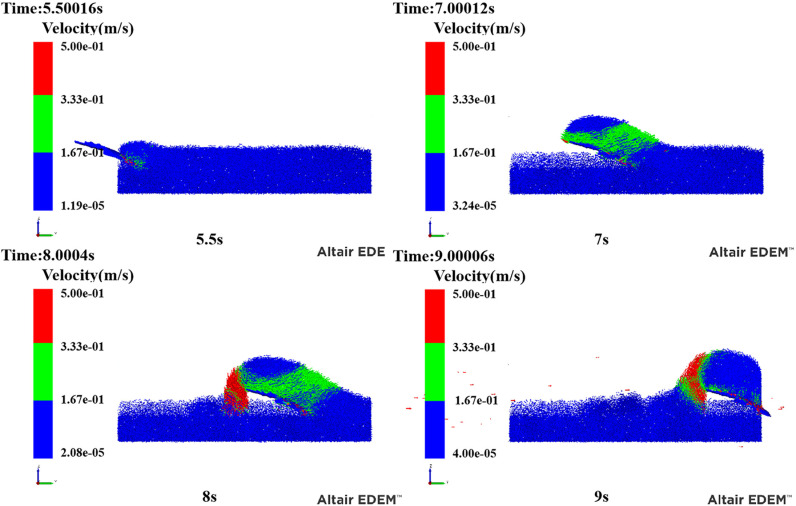
Simulation of analogue images with different time steps. Movie in S3 Movie.

Three-dimensional modelling software was used to model the three types of excavation shovels to be imported into EDEM, and the models are shown in Fig 8. The simulated images for different time steps of the simulation are shown in Fig 9.

#### Influence of factors on digging resistance.

In the process of analysing the digging resistance, the drag reduction rate is an important index to measure the drag reduction performance of the digging shovel [24]. The drag reduction effect is reflected by the drag reduction rate, and the formula for the drag reduction rate is


$$\eta =\frac{F_{x}-F_{y}}{F_{x}}$$ (14)

where: *F*_x_ -- digging resistance of comparison digging shovel, *F*_y_ -- digging resistance of bionic digging shovel.

In accordance with agronomic requirements, the shovel’s digging depth was determined to be 200 mm. It is commonly known that potato seeding is carried out through ridging, with a seeding depth of 70 to 100 mm. A digging depth of 200 mm ensures the integrity of the potato pieces and enhances soil crushing efficiency, thereby aiding in reducing tillage resistance once harvesting is completed.

**Fig 10:**
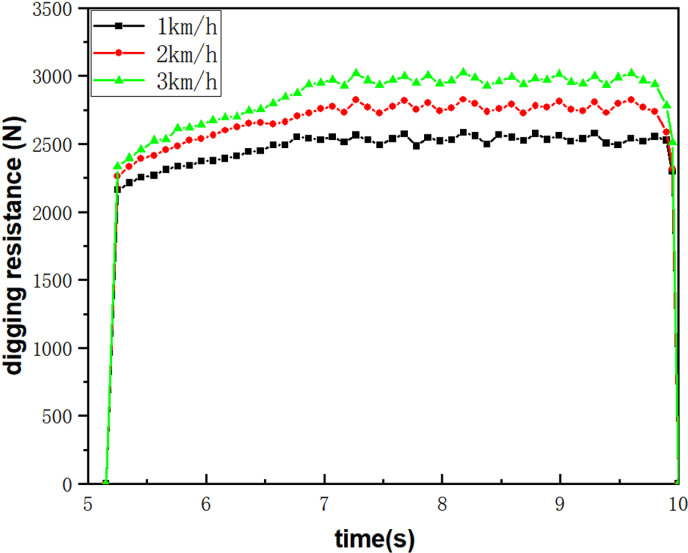
Average digging resistance at different forward speeds. Table in S1 Table.

The bionic digging shovel was imported into EDEM for simulation tests. The unit of digging speed used in this paper is meters per second (m/s). Based on literature reviews, potato harvesters operate at forward speeds ranging from 0.27 to 0.83 m/s [25]. Therefore, we selected forward speeds for the digging shovel at 0.27 m/s, 0.56 m/s, and 0.83 m/s. Using a post-processing tool (Analyst), the average resistance of the digging shovel throughout the entire working process was extracted, focusing on the digging resistance values between 5.3 and 10 seconds during the actual action time period. The corresponding curves were fitted using Origin 2021 software, and the stable working stage was chosen to extract the average excavation resistance per unit time, as illustrated in Fig 10. Calculations show that the average resistance for forward speeds of 0.27, 0.56, and 0.83 m/s are 2475.38N, 2678.26N, and 2845.79N.

**Fig 11:**
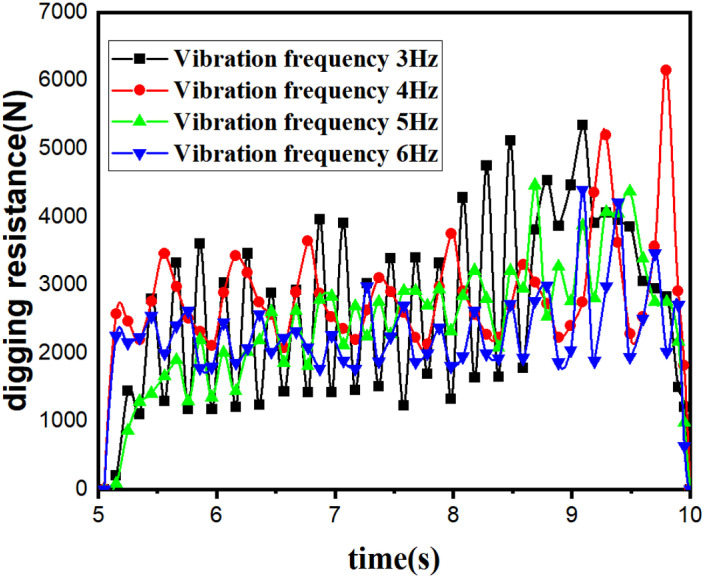
Digging resistance at different vibration frequencies. Table in S2 Table.

Subsequently, the vibration frequency of the bionic excavation shovel is analysed. The parameter is set to a forward speed of 0.27m/s and the rest of the parameters are the same as above. and it is known from the information that the vibration frequency of potato vibration excavation is between 3–6 Hz [26], therefore, it is chosen to simulate the vibration frequency of 3Hz, 4Hz, 5Hz, and 6Hz, and to determine the effect of the vibration frequency on the digging resistance, and the digging resistance of the different vibration frequencies is shown in Fig 11. Calculations show that the excavation resistance at vibration frequencies of 3, 4, 5 and 6 Hz are 2725.06N, 2892.47N, 2550.23N and 2330.12N respectively.

**Fig 12:**
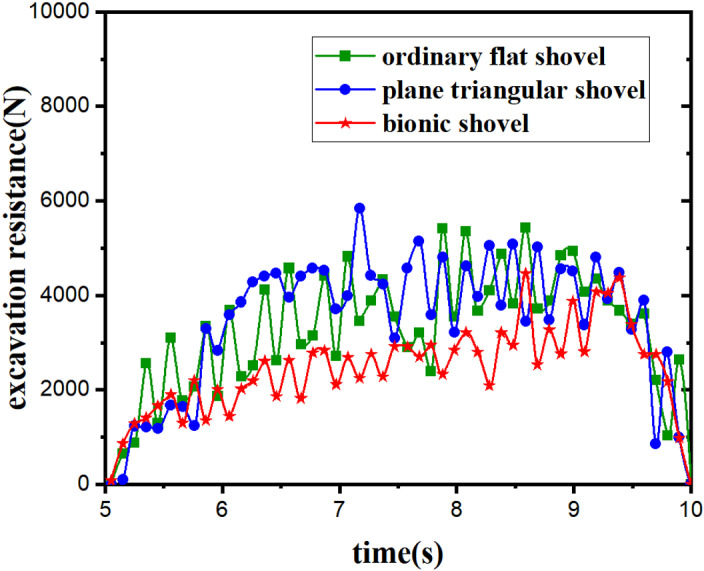
Resistance curves of three types of excavators. Table in S3 Table.

Three kinds of excavation shovels were imported into the EDEM. Based on the results of the aforementioned two sets of simulation tests, it has been concluded that digging resistance increases with an increase in digging speed and decreases with an increase in vibration frequency. Therefore, taking into account relevant agronomic requirements, the parameters were set to a digging speed of 0.27 m/s and a vibration frequency of 6 Hz. Fig 12 illustrates the digging resistance of the three types of digging shovels under these conditions.

During the working process, the digging resistance of the three types of digging shovels fluctuated in a regular pattern. Notably, the triangular shovel demonstrated the highest peak in resistance, whereas the bionic shovel exhibited relatively smaller fluctuations. In terms of average digging resistance, the bionic shovel recorded the lowest value of 2330.12N, compared to 3068.38N for the ordinary flat shovel and 3362.43N for the triangular shovel. This suggests that, under identical working conditions, the bionic shovel encounters the least amount of average digging resistance.

The simulation analogue test of digging shovel based on EDEM discrete element design embodies the drag reduction performance of bionic digging shovel from the theoretical point of view, and the digging resistance increases with the increase of the forward speed and shows a decreasing trend with the increase of the vibration frequency. It provides a reference basis for the subsequent field test.

### Field validation trials

In order to further verify the resistance reduction characteristics of the bionic digging shovel, a field verification test was carried out in August 2024. The test field of Shaozhuang Town Agricultural Machinery Test Field of Shandong Flint Agricultural Science and Technology Co., Ltd. was selected as the test site. The five-point sampling method was used to test three groups of data to get the average value of the test plot’s soil firmness and soil moisture, and soil firmness was 450.95kPa, and soil moisture content was 55.5%.

**Fig 13:**
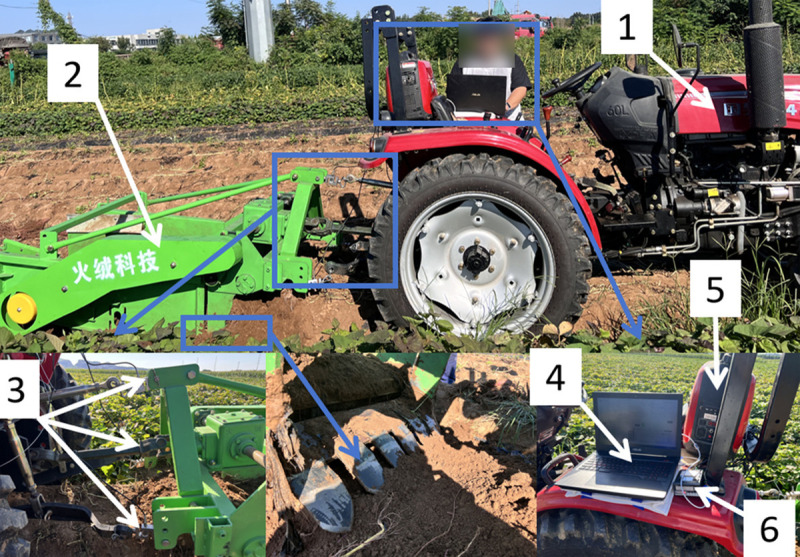
Preparation of the test setup. 1. Dongfanghong ME704-N Tractor 2. 4USY-90 Potato Harvester 3. ZNL Transmission ZNLBS-G-1T S-Type Tension Sensor 4. Data Output Equipment 5. Portable Petrol Generator 6. ZNL Transmission ZN5X-485 Data Processor.

The digging resistance test system of this test consists of Dongfanghong ME704-N tractor, 4USY-90 potato harvester, three ZNLBS-G-1T S-type tension transducers of ZNL Transmission, data output equipment, portable petrol generator, and ZNL Transmission ZN5X-485 data processor. The test setup is shown in Fig 13. S1 and S2 Movies are provided. The photos of the excavation experiments are displayed in S1 and S2 Figs.

In order to further investigate the effect of bionic digging shovel on digging resistance, this test adopts three factors and three levels of orthogonal combination test by using operating speed, different shovel shapes and vibration frequency as test factors, and the test index is digging resistance, and the test factor table is shown in Table 2. Select the same plot of different ridges for the test, from the tractor start to finish as 1 test, the forward length is 10 meters, using force measuring device for digging resistance detection, data analysis method as above. A total of 17 groups were tested, and the field test was conducted according to the 17 groups of data in Table 3 below.

**Table 2:** Table of test factors.

Encodings	Experimental factors		
	Operating speed A (m/s)	Spatula B	Vibration frequency C (Hz)
(−1)	0.27	bionic shovel	4
(0)	0.56	ordinary flat shovel	5
(+1)	0.83	plane triangular shovel	6

**Table 3:** Test protocol and results.

Number	Experimental factors			Digging resistance (N)
	Operating speed A (m/s)	Spatula B	Vibration frequency C (Hz)	
1	−1	1	0	3820.21
2	0	0	0	3798.12
3	1	0	1	3854.21
4	0	0	0	3811.53
5	0	0	0	3781.53
6	0	0	0	3758.55
7	0	−1	−1	3682.69
8	−1	0	−1	3693.55
9	0	1	−1	4289.77
10	0	−1	1	3621.22
11	1	0	−1	4155.5
12	0	1	1	3957.84
13	1	1	0	4319.45
14	−1	−1	0	3511.53
15	0	0	0	3754.99
16	1	−1	0	3800.5
17	−1	0	1	3704.96

### Analysis of test results

**Fig 14:**
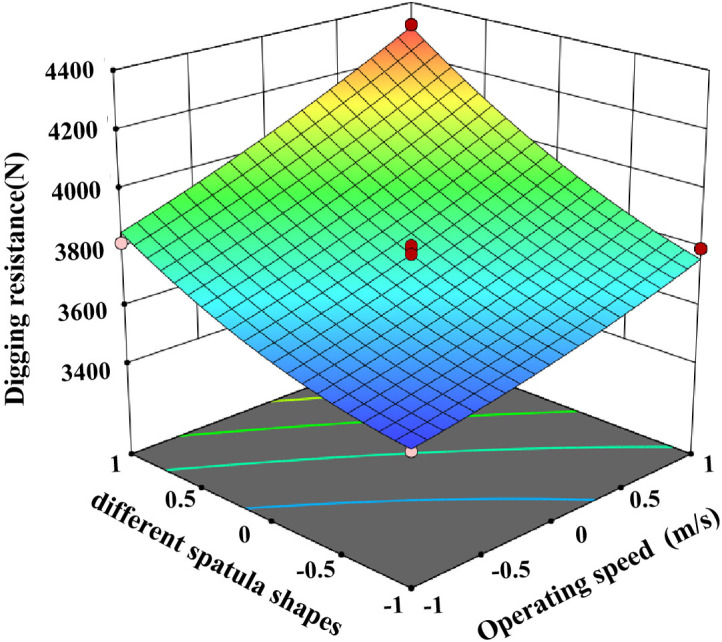
Influence of operating speed and shovel shape on resistance.

**Fig 15:**
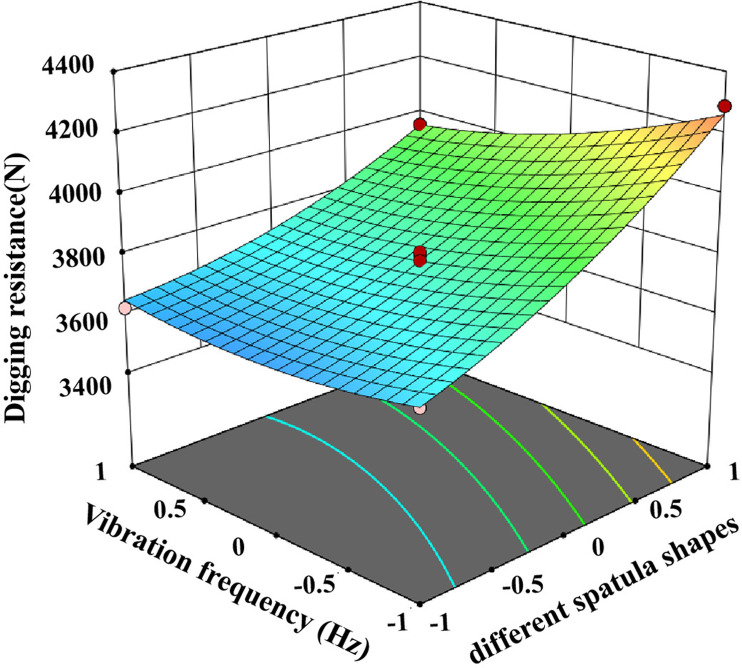
Effect of operating speed and vibration frequency on resistance.

**Fig 16:**
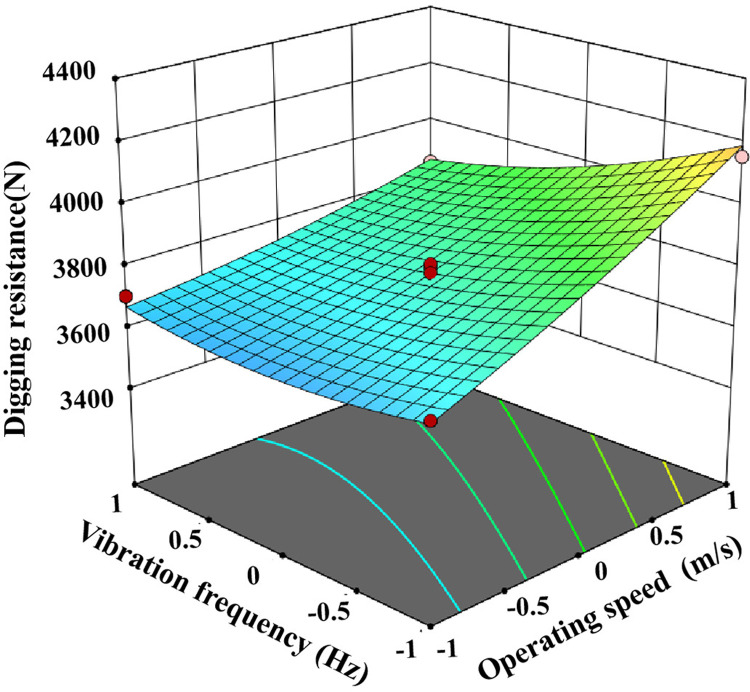
Effect of different shovel shape and vibration frequency on drag force.

The test data were imported into Design expert 13 for analysis and the ANOVA is shown in Table 4. The ANOVA of Table 4 shows that the P-value of the index digging resistance of this test is less than 0.01, and the P-value of the misfit term is greater than 0.05, indicating that the regression equation is highly significant and well fitted [27]. Therefore this regression equation can be used to optimally analyse the operating parameters of potato bionic digging shovels under clayey soil conditions, and the interaction factors on digging resistance are shown in Figs 14–16.

**Table 4:** Analysis of variance of the regression equation.

Origin	Digging resistance (N)				
	Source	Sum of squares	Mean square	F-value	P-value
model	778400	9	86483.76	64.77	< 0.0001^**^
A	244800	1	244800	183.34	< 0.0001^**^
B	392200	1	392200	293.75	< 0.0001^**^
C	58358.94	1	58358.94	43.71	0.0003^**^
AB	11053.37	1	11053.37	8.28	0.0237^*^
AC	24445.32	1	24445.32	18.31	0.0037^**^
BC	18287.15	1	18287.15	13.70	0.0076^**^
A^2^	2242.26	1	2242.26	1.68	0.2361
B^2^	14608.07	1	14608.07	10.94	0.0130^*^
C^2^	9714.90	1	9714.90	7.28	0.0308^*^
Residual	9346.11	7	1335.16		
Lack of Fit	6940.15	3	2313.38	3.85	0.1130
Pure Error	2405.96	4	601.49		
Cor Total	787700	16			

* indicates significant differences (P < 0.05).

** indicates highly significant differences (P < 0.01).

In the regression model for digging resistance, the effects of A, B, C, AC and BC on the model were highly significant (p < 0.01), AB, B^2^ and C^2^ were significant, and the effects of the other factors were not significant. By incorporating the regression squares and degrees of freedom of the non-significant interaction terms into the residual terms, the regression equation for digging resistance is:


$$Y_{1}=3780.94+174.93A+221.42B-85.41C-78.17AC-67.62BC+52.57AB+58.9B^{2}+48.03C^{2}$$ (15)

The digging resistance of the excavating shovel is closely related to the energy consumption, the greater the resistance, the higher the energy consumption [28]. Table 4 shows that the F-value of the influence of each factor on the digging resistance is 183.34 for the operation speed, 293.75 for the shovel shape, 43.71 for the vibration frequency, and the primary and secondary relationship is shovel shape > forward speed > vibration frequency, and the optimal scheme is A_−1_B_−1_C_1_ through the extreme difference analysis. different shovel shapes have the biggest influence on the digging resistance, followed by forward speed, and finally vibration frequency, and the optimal scheme is A_−1_B_−1_C_1_ through the analysis. A_−1_B_−1_C_1_ was selected for the clay soil, i.e., the shovel shape was selected as bionic excavation shovel, the operating speed was 1km/h, and the vibration frequency was 6Hz.

### Experimental verification

Validation was carried out for the conclusions drawn from the above orthogonal combination tests. Ten sets of validation tests were conducted using the digging resistance during potato harvesting under sticky soil conditions as the test index. Under the conditions of operating speed of 0.27m/s and vibration frequency of 6Hz, the ordinary flat shovel (1), Plane triangular shovel (2) and bionic shovel (3) were tested for the comparison of digging resistance. The test method is consistent with the orthogonal combination tests, and the test results are shown in Table 5.

**Table 5:** Comparison test results.

Project	Digging resistance (N)		
	Ordinary flat shovel	Plane triangular shovel	Bionic shovel
1	4216.43	4421.45	3421.64
2	4509.82	4625.59	3758.23
3	4387.56	4587.36	3574.59
4	4454.35	4700.14	3702.98
5	4222.99	4536.87	3483.15
6	4510.77	4685.92	3698.42
7	4378.21	4512.63	3625.77
8	4409.68	4601.48	3534.88
9	4355.44	4499.75	3729.34
10	4483.14	4610.84	3599.58

One-way ANOVA was used to analyse the Digging resistance of different shovel shapes using SPSS software and LSD method was used in multiple comparisons. The results showed that there was a significant difference between different shovel shapes in terms of digging resistance at a set significance level of 0.05 (P < 0.001). Specifically, the average digging resistance of Ordinary flat shovel (1) was 4392.84 N, the digging resistance of planar triangular shovel (2) was 4578.20 N, and the digging resistance of bionic shovel (3) was 3612.86 N. The difference between 1 and 3 was 779.98 N, and the difference between 2 and 3 was 965.35 N, as shown in Table 6. The results show that the bionic shovel reduces the resistance by 17.76% relative to the ordinary flat shovel and 21.09% relative to the Plane triangular shovel.

**Table 6:** Multiple comparisons.

(I) Project	(J) Project	Mean difference (I–J)	Std. error	Sig.
1	2	−185.36*	45.54	<0.001
	3	779.98*	45.54	<0.001
2	1	185.36*	45.54	<0.001
	3	965.35*	45.54	<0.001
3	1	−779.98*	45.54	<0.001
	2	−965.35*	45.54	<0.001

* The mean difference is significant at the 0.05 level.

Due to the simulation test process, in order to speed up the simulation speed, the imported model is the digging shovel and its connecting cross plate, the whole machine is not imported, and the same part is omitted. Therefore, there are differences in the digging resistance when the results are displayed. However, this test aims to investigate the drag reduction effect of different excavating shovels, and verify that the order of digging resistance of different excavating shovels in the test is the same as the order of resistance size in the simulation test, and the drag reduction effect is similar.

## Conclusions

A streamlined potato harvesting digging shovel modeled after a catfish head is designed for heavy soil conditions. The front end of the digging shovel is bionically designed by fitting the curved curve of the catfish mouth, the ridges and wefts of the head. The material is selected as 65Mn, and the installation method is a transverse homogeneous arrangement. Force analysis is carried out for the face of the digging shovel and the potato-soil mixture, considering the angle of the digging shovel’s entry into the soil. The forward speed and the vibration frequency are the main factors influencing this digging angle of entry, and they are also the primary factors affecting the digging resistance. The optimum angle of entry has been calculated to be 15°.

Using EDEM to analyse different shovel shapes, forward speeds and vibration frequencies, and taking the working resistance of the digging process as a test index. The resistance reduction performance of the bionic digging shovel is theoretically demonstrated, and the digging resistance increases with the increase of forward speed, and decreases with the increase of vibration frequency. It provides a reference basis for the subsequent field test.

Further investigate the effect of bionic digging shovel on digging effect through field test, orthogonal test results show that the primary and secondary relationship of each factor on digging resistance are shovel shape > forward speed > vibration frequency. The optimal solution was confirmed to be the bionic digging shovel with the shape of the shovel, the operating speed of 0.27 m/s, and the vibration frequency of 6 Hz. bionic digging shovel resistance was 3612.86N, and the resistance of the bionic shovel was reduced by 17.76% relative to the ordinary flat shovel, and 21.09% relative to the plane triangle shovel.

Compared with the traditional digging shovel, the bionic catfish head curve type digging shovel has a better drag reduction effect under the same operating conditions. It meets the design requirements of the potato digging shovel to reduce the drag and increase the efficiency, and provides a theoretical basis and design foundation for the subsequent potato digging machinery to reduce the drag of the whole machine.

## Supporting information

S1 Movie Layout of excavation equipment and sensor devices. (MP4)

S2 Movie Experimental work video. (MP4)

S3 Movie Simulated movie for emulation. (MP4)

S1 Fig Front display of the digging equipment. (JPG)

S2 Fig Side display of the digging equipment. (JPG)

S1 Table Data on average digging resistance at different forward speeds. (XLSX)

S2 Table Data on digging resistance at different vibration frequencies. (XLSX)

S3 Table Data on resistance curves of three types of excavators. (XLSX)
