# Supplementary figures and images for: Design and experiment of a bionic drag-reducing digger for tuberous crops under heavy soil conditions
Source: PLoS One. 2025 Feb 25;20(2):e0318526. doi: 10.1371/journal.pone.0318526 (PMC11856321; doi:10.1371/journal.pone.0318526)

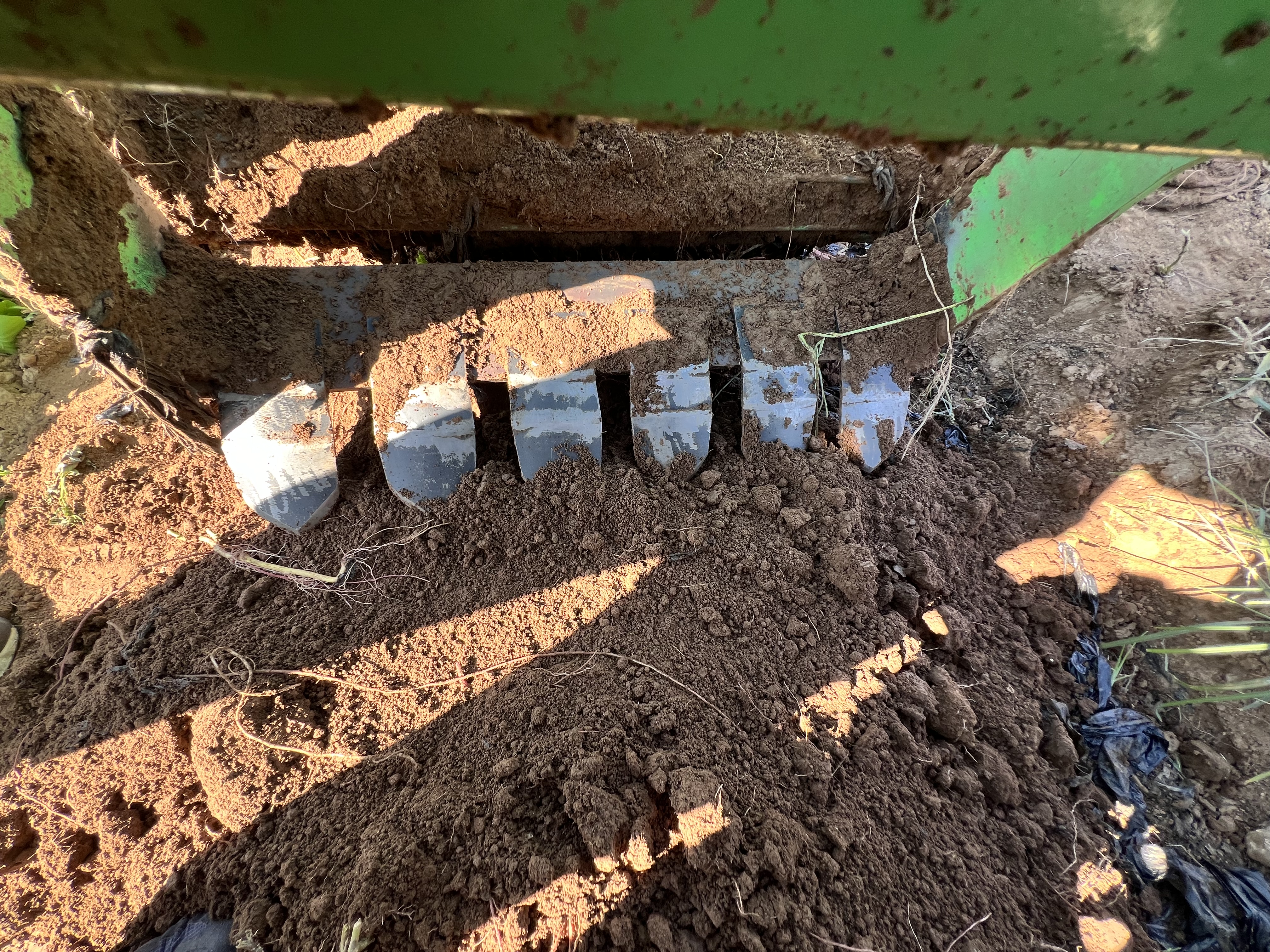

Supplement: S1 Fig — (JPG) [file pone.0318526.s004.jpg]

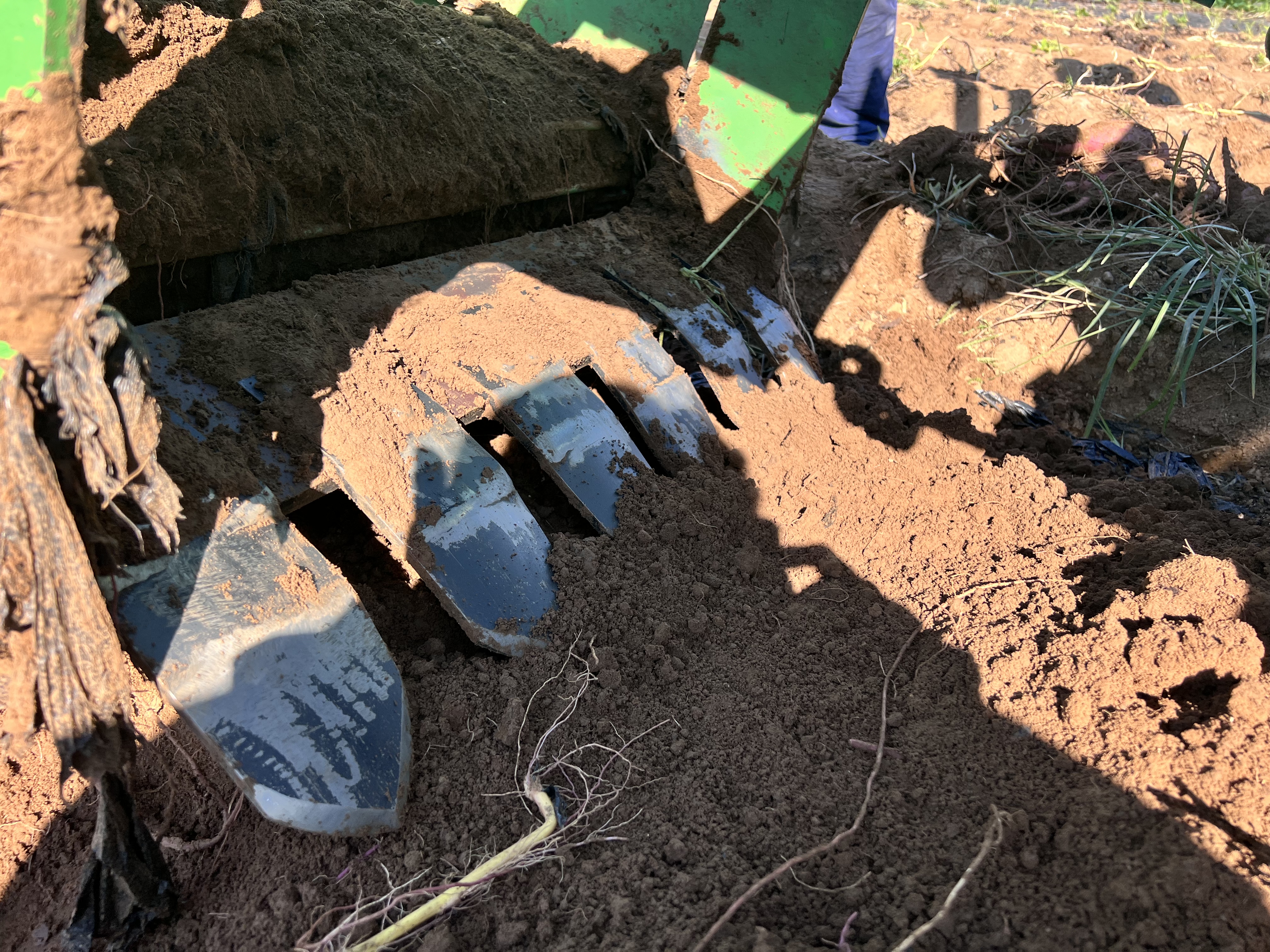

Supplement: S2 Fig — (JPG) [file pone.0318526.s005.jpg]
